# Association of Different Prescribing Patterns for Oral Corticosteroids With Fracture Preventive Care Among Older Adults in the UK and Ontario

**DOI:** 10.1001/jamadermatol.2023.2495

**Published:** 2023-08-09

**Authors:** Julian Matthewman, Mina Tadrous, Kathryn E. Mansfield, Deva Thiruchelvam, Donald A. Redelmeier, Angela M. Cheung, Iliana C. Lega, Daniel Prieto-Alhambra, Lawrence A. Cunliffe, Amy Mulick, Alasdair Henderson, Sinéad M. Langan, Aaron M. Drucker

**Affiliations:** 1 Department of Non-Communicable Disease Epidemiology, London School of Hygiene & Tropical Medicine, London, United Kingdom; 2 Women’s College Research Institute, Women’s College Hospital, Toronto, Canada; 3 Leslie Dan School of Pharmacy, University of Toronto, Toronto, Canada; 4 ICES (previously known as Institute for Clinical Evaluative Sciences), Toronto, Canada; 5 Department of Medicine, University of Toronto, Toronto, Canada; 6 Independent Patient Partner, Canada

## Abstract

**Importance:**

Identifying and mitigating modifiable gaps in fracture preventive care for people with relapsing-remitting conditions such as eczema, asthma, and chronic obstructive pulmonary disease who are prescribed high cumulative oral corticosteroid doses may decrease fracture-associated morbidity and mortality.

**Objective:**

To estimate the association between different oral corticosteroid prescribing patterns and appropriate fracture preventive care, including treatment with fracture preventive care medications, among older adults with high cumulative oral corticosteroid exposure.

**Design, Setting, and Participants:**

This cohort study included 65 195 participants with UK electronic medical record data from the Clinical Practice Research Datalink (January 2, 1998, to January 31, 2020) and 28 674 participants with Ontario, Canada, health administrative data from ICES (April 1, 2002, to September 30, 2020). Participants were adults 66 years or older with eczema, asthma, or chronic obstructive pulmonary disease receiving prescriptions for oral corticosteroids with cumulative prednisolone equivalent doses of 450 mg or higher within 6 months. Data were analyzed October 22, 2020, to September 6, 2022.

**Exposures:**

Participants with prescriptions crossing the 450-mg cumulative oral corticosteroid threshold in less than 90 days were classified as having high-intensity prescriptions, and participants crossing the threshold in 90 days or more as having low-intensity prescriptions. Multiple alternative exposure definitions were used in sensitivity analyses.

**Main Outcomes and Measures:**

The primary outcome was prescribed fracture preventive care. A secondary outcome was major osteoporotic fracture. Individuals were followed up from the date they crossed the cumulative oral corticosteroid threshold until their outcome or the end of follow-up (up to 1 year after index date). Rates were calculated for fracture preventive care and fractures, and hazard ratios (HRs) were estimated from Cox proportional hazards regression models comparing high- vs low-intensity oral corticosteroid prescriptions.

**Results:**

In both the UK cohort of 65 195 participants (mean [IQR] age, 75 [71-81] years; 32 981 [50.6%] male) and the Ontario cohort of 28 674 participants (mean [IQR] age, 73 [69-79] years; 17 071 [59.5%] male), individuals with high-intensity oral corticosteroid prescriptions had substantially higher rates of fracture preventive care than individuals with low-intensity prescriptions (UK: 134 vs 57 per 1000 person-years; crude HR, 2.34; 95% CI, 2.19-2.51, and Ontario: 73 vs 48 per 1000 person-years; crude HR, 1.49; 95% CI, 1.29-1.72). People with high- and low-intensity oral corticosteroid prescriptions had similar rates of major osteoporotic fractures (UK: crude rates, 14 vs 13 per 1000 person-years; crude HR, 1.07; 95% CI, 0.98-1.15 and Ontario: crude rates, 20 vs 23 per 1000 person-years; crude HR, 0.87; 95% CI, 0.79-0.96). Results from sensitivity analyses suggested that reaching a high cumulative oral corticosteroid dose within a shorter time, with fewer prescriptions, or with fewer or shorter gaps between prescriptions, increased fracture preventive care prescribing.

**Conclusions:**

The results of this cohort study suggest that older adults prescribed high cumulative oral corticosteroids across multiple prescriptions, or with many or long gaps between prescriptions, may be missing opportunities for fracture preventive care.

## Key Points

**Question:**

Are prescribing patterns for older people receiving high cumulative doses of oral corticosteroids associated with adequate fracture preventive care?

**Findings:**

This cohort study of 65 195 older adults in the UK and 28 674 older adults in Ontario, Canada, found that individuals who were prescribed high cumulative oral corticosteroid doses gradually or intermittently across multiple prescriptions were about half as likely as individuals prescribed a similar dose in 1 prescription or within a short period of time to receive guideline-indicated fracture preventive care.

**Meaning:**

Increasing attention to individuals receiving prescriptions for high cumulative oral corticosteroid doses discontinuously may help close an identified gap in fracture preventive care.

## Teaser

This cohort study conducted in the UK and Ontario, Canada, assesses the association between oral corticosteroid prescribing patterns and appropriate fracture preventive care among older adults with high cumulative oral corticosteroid exposure.

## Introduction

Oral corticosteroids are a major cause of secondary osteoporosis and subsequent fractures.^1,2,3^ Older people are particularly vulnerable.^4^ People using oral corticosteroids for 3 or more months at a prednisolone equivalent dose of 5 mg daily or higher (corresponding to a cumulative prednisolone equivalent dose threshold of 450 mg) should be considered at increased risk of fracture, and depending on other risk factors, such as age, should be referred for bone mineral density measurements and/or treated with fracture preventive care medications, such as bisphosphonates.^5^ This guidance is reflected in the commonly used FRAX fracture risk assessment tool,^6,7^ which is recommended in UK,^8^ Canadian,^9^ and US^10^ guidelines.

Rather than providing a single prescription, or a small number of prescriptions sequentially without gaps (as might be the case for the long-term treatment of rheumatoid arthritis),^11^ oral corticosteroids are often prescribed in short discontinuous bursts to treat disease flares of relapsing-remitting conditions, such as eczema,^12^ asthma,^13^ and chronic obstructive pulmonary disease (COPD).^14^ We hypothesized that treating physicians’ awareness of patients crossing a cumulative oral corticosteroid dose threshold is lower in the latter type of prescriptions to treat COPD, eczema, asthma, which would constitute a modifiable gap in fracture preventive care. Since these relapsing-remitting conditions are often managed by different specialist and generalist physicians, including dermatologists, respirologists, internists, family physicians, and emergency physicians, that gap in care would be relevant to many clinical settings. Identifying and mitigating that gap could reduce the high morbidity and mortality associated with fractures.^15^

The objective of this study was to determine whether oral corticosteroid prescribing patterns were differentially associated with receiving guideline-recommended fracture preventive care among older adults with eczema, asthma, or COPD.

## Methods

### Study Design and Setting

We conducted parallel cohort studies using routinely collected UK general practice data (January 2, 1998, to January 31, 2020) and Ontario, Canada, population-based administrative health data (April 1, 2002, to September 30, 2020) (Figure). The UK study was approved by the Independent Scientific Advisory Committee, the London School of Hygiene & Tropical Medicine Research Ethics Committee, and the Clinical Practice Research Datalink (CPRD) Independent Scientific Advisory Committee. For the Ontario study, ICES (previously Institute for Clinical Evaluative Sciences) is a prescribed entity under section 45 of Ontario’s Personal Health Information Protection Act. The use of data held at ICES is authorized under section 45 of Ontario’s Personal Health Information Protection Act and does not require review by a research ethics board. This project was conducted under section 45 and approved by the ICES Privacy and Legal Office. The need to obtain informed consent was waived because all data were deidentified (UK) or were population-based administrative data (Ontario). This study followed the Reporting of Studies Conducted Using Observational Routinely-Collected Data for pharmacoepidemiology (RECORD-PE) reporting guideline (eAppendix in Supplement 1).

**Figure.**
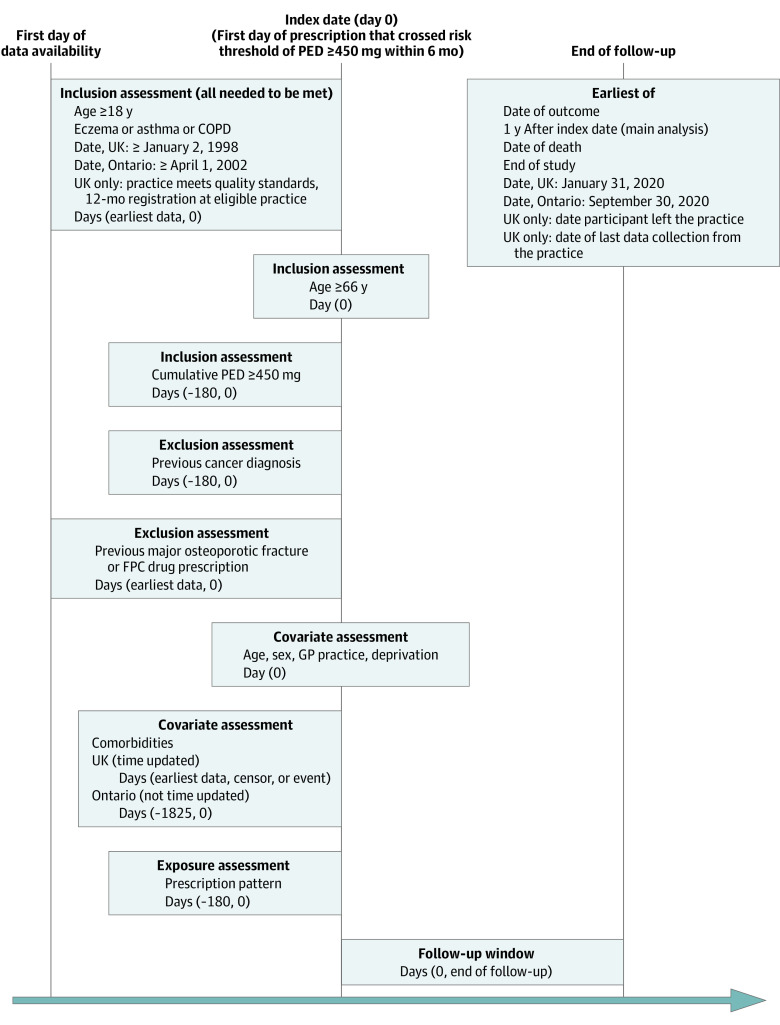
Study Design Source population for the UK study was all people attending general practitioners (GPs) in the UK, and for the Ontario study, all people attending GPs in Ontario, Canada. Database population for the UK was individuals included in the Clinical Practice Research Datalink, and for the Ontario study, individuals included in the ICES database. Study population was people with eczema, asthma, or chronic obstructive pulmonary disease (COPD) who should be considered for fracture preventive care (FPC) (ie, individuals with prescriptions that crossed the risk threshold of a 450-mg prednisolone equivalent dose [PED]).

### Data Sources

The UK study used deidentified primary care data from CPRD GOLD, which includes more than 11 million people from 674 practices in the UK^16^ linked to deprivation data (Carstairs index)^17^ and Office for National Statistics death data (linkages provided directly through CPRD). The Ontario study used population-based primary and secondary care administrative data from ICES, with linkages between multiple databases (eMethods 1 in Supplement 1).

### Study Population

We identified cohorts of people with eczema, asthma, or COPD. In the UK, we included all individuals with at least 1 diagnostic code for eczema, asthma, or COPD, and in Ontario, we included people with at least 1 physician visit for eczema, presence of at least 1 hospitalization or 2 or more physician visits in a 2-year period for asthma,^18^ and at least 1 hospitalization or at least 1 physician visit for COPD.^19^ From these identified people, we selected a subset of adults 66 years of age or older crossing the cumulative oral corticosteroid dose high-risk threshold of 450 mg of the prednisolone equivalent dose in the last 6 months (eTable 6 in Supplement 1). According to FRAX recommendations, all of these people should be considered for fracture preventive care.^6^ The index date was the start date of the first prescription that would surpass the risk threshold for that individual. We excluded people who previously received a fracture preventive care drug, experienced a major osteoporotic fracture, or showed evidence of receiving a cancer diagnosis in the previous 6 months (to identify actively treated cancers). Individuals could be included only once.

### Exposures, Outcomes, and Covariates

#### Corticosteroid Prescribing Patterns

We used information on dose and duration of oral corticosteroid prescriptions from cleaned prescription data (eMethods 2 in Supplement 1). We ascertained the time taken to reach the risk threshold (ie, cumulative prednisolone equivalent dose of 450 mg in 6 months), the number of gaps between prescriptions within that period, and the total length of these gaps.

For the primary exposure, we classified individuals as having low-intensity (≥90 days to cross the risk threshold) vs high-intensity (<90 days to cross the risk threshold) oral corticosteroid prescriptions (eFigure 1 in Supplement 1). In sensitivity analyses, we (1) defined exposure based on the number and total length of gaps between prescriptions, (2) used different cut points to classify exposure, and (3) used a log-transformed continuous exposure definition (Table 1).

**Table 1.** Exposure Definition, Study Cohort, and Model Covariate Changes in Sensitivity Analyses

Sensitivity analysis^a^	Justification
Exposure definition change^b^	
Use time to risk threshold (0 vs 1-180 d)	Prescribing a dose crossing the 450-mg prednisolone equivalent dose risk threshold in a single prescription may influence the decision to prescribe fracture preventive care.
Use total length of gaps (0-89 vs 90-180 d)	Lengths of gaps between prescriptions may influence awareness of previously prescribed cumulative doses.
Use total length of gaps (0 vs 1-180 d)	Prescribing a dose crossing the 450-mg prednisolone equivalent dose risk threshold in a single prescription may influence the decision to prescribe fracture preventive care.
Use No. of gaps (0-1 vs ≥2 gaps)	Number of gaps between prescriptions may influence awareness of previously prescribed cumulative doses.
Use No. of gaps (0 vs ≥1 gaps)	Prescribing a dose crossing the 450-mg prednisolone equivalent dose risk threshold in a single prescription may strongly influence the decision to prescribe fracture preventive care.
Use log-transformed continuous variable of log_10_ (days to risk threshold + 1)	Likelihood of receiving fracture preventive care may decrease rapidly at first and then slow with the No. of days to reach risk threshold. Estimate hazard ratios with the exposure as a continuous variable by log_10_-transformed No. of days to reach risk threshold (+ 1 to avoid a zero value at 0 d)
Cohort composition change^c^	
Follow-up not limited	Effect of (missed opportunities for) fracture preventive care is likely to occur over a longer period of time; therefore, considered analyses with follow-up time not limited to 1 y as the main analyses for the fracture outcome.
	For fracture preventive care outcome, performed these analyses as sensitivity analyses.
UK only: age not limited to ≥66 y, ie, all adults ≥18 y are eligible	Fracture preventive care drugs are rarely prescribed to younger individuals; however, it may be appropriate to include individuals of all ages to not miss special cases in which fracture preventive care is prescribed to younger individuals.
UK only: use different method to clean oral corticosteroid prescription information with more values imputed (for prescription quantity information, in addition to values that were recorded as missing, values that were recorded as 0 were imputed).	Data cleaning of oral corticosteroid prescription data alters the cohort composition. Oral corticosteroid prescriptions with 0 recorded as the quantity may be prescribed “as needed” or may constitute cases in which true quantity is not entered.
Model covariate change^d^	
Adjust for age group, sex, and deprivation	Although cohort was selected by age (≥66 y), there may still be differences in age, sex, and deprivation between groups with high- vs low-intensity prescriptions.
Adjust for age group, sex, deprivation, eczema, asthma, COPD, and rheumatoid arthritis	Groups differed in disease status, which may be associated with the rate of fracture preventive care prescribing or fractures.
Ontario only: covariates of the main analysis, rurality, dementia, drugs decreasing fracture risk, drugs increasing fracture risk, inhaled or nasal corticosteroids, injectable corticosteroids, topical corticosteroids, other corticosteroids, oral corticosteroid in the year prior to cohort entry, health care use in the year prior to cohort entry (physician visits [0-12, ≥13], hospitalization [yes, no], No. of physicians prescribing oral corticosteroid [1, ≥2]), specialty of physician prescribing oral corticosteroid (family practice, dermatology, emergency medicine, and other)	Other variables may confound the association between oral corticosteroid prescribing pattern and fracture preventive care or fractures.

Abbreviation: COPD, chronic obstructive pulmonary disease.

^a^ For both Ontario and UK analyses unless otherwise stated.

^b^ Exposure definition for main analysis in both UK and Ontario cohorts defined using time to risk threshold as high (0-89 days) vs low (90-180 days) intensity.

^c^ Cohort composition for main analysis in both UK and Ontario cohorts fracture preventive care outcome had follow-up time limited to 1 year, with follow-up time not limited for fracture outcome. People were included only if they were aged 66 years or older. For UK only, due to missing information in electronic health records, values for quantity and daily dose of prescriptions that were recorded as missing were imputed.

^d^ Models not adjusted for any covariates in main analysis in both UK and Ontario cohorts.

#### Fracture Preventive Care

Our primary outcome was prescriptions for fracture preventive care medications, which are recommended in guidelines for this patient population (including bisphosphonates, bazedoxifene, burosumab, raloxifene, and teriparatide).^10^ For a secondary outcome, we expanded the definition to include either prescriptions for fracture preventive medications or bone mineral density measurements (dual-energy x-ray absorptiometry; DEXA). In another secondary analysis for the UK cohort only, we used any calcium or vitamin D prescription as a secondary outcome definition.

#### Major Osteoporotic Fractures

Major osteoporotic fractures were a secondary outcome. For the UK cohort, we identified fractures of spine, hip (proximal femur), wrist, or pelvis, excluding surgical or cancer-related fractures, in primary care morbidity coding. For the Ontario cohort, we identified hip, vertebral, and humerus and forearm fractures using standardized administrative algorithms.^20^

#### Negative Control Outcomes

Negative control outcomes are outcomes that are known not to be associated with exposure but share the same potential sources of bias with the primary outcome.^21^ For negative control outcomes, we included prescriptions for epilepsy and migraine medications (UK) as drugs that should not be associated with oral corticosteroid prescribing, and drugs used for anxiety (UK and Ontario) as medications potentially associated with oral corticosteroid prescribing but that should not be associated with the pattern of oral corticosteroid prescribing.

#### UK Covariates

We identified age and sex at the index date. All individuals had at least 1 of eczema, asthma, or COPD to be eligible for inclusion. Eczema, asthma, and COPD were also defined as time-updated variables with status changing on first record. That is, people were considered as not having the disease until the first record of an appropriate primary care diagnostic code. We also identified comorbid rheumatoid arthritis. As a measure of socioeconomic status, we used quintiles of the Carstairs index of material deprivation (at the individual level if available, otherwise at the practice level) from 2011.

#### Ontario Covariates

We obtained individuals’ age, sex, and home location at the index date. We identified eczema, asthma, COPD, rheumatoid arthritis, and dementia during a 5-year look back, which required at least 2 physician visits with the diagnosis. We identified medications that may increase or decrease fracture risk, including other types of corticosteroids (inhaled or nasal, injectable, topical, and other) used in the year prior to the index date. We identified health care use during the year prior to the index date, including the number of physician visits and hospital visits. We established the specialty of the physician, or physicians prescribing any oral corticosteroid that contributed to crossing the risk threshold. As a measure of socioeconomic status, we used quintiles based on neighborhood income.

### Statistical Analysis

We calculated descriptive statistics, including participant counts and distribution of characteristics by oral corticosteroid prescribing pattern exposure status.

Individuals were followed up until they either experienced an outcome (fracture preventative care or fracture) or were censored (earliest of 1 year after index date [main analysis], death, left practice [UK only], last data collection from the practice [UK only], or end of the study [UK, January 31, 2020; Ontario, September 30, 2020]). We limited follow-up to 1 year after index date for the fracture preventive care outcome so that any prescriptions for fracture preventive care would be associated with crossing the risk threshold of 450-mg cumulative prednisolone equivalent dose. Since both a detrimental association of oral corticosteroid use with bone health and a beneficial association of oral corticosteroid use with fracture preventive care may take longer than 1 year to occur, we did not limit the follow-up time in analyses for the fracture outcome.

We calculated crude rates and estimated hazard ratios (HRs), with follow-up time as the underlying timescale, for the association between oral corticosteroid prescribing pattern and the outcome using Cox proportional hazards regression analysis. We fitted crude models, and models adjusted for age, sex, deprivation, eczema, asthma, COPD, and rheumatoid arthritis (which is included as a risk factor in FRAX and is treated with oral corticosteroids). In Ontario, we additionally fit models adjusting for all other covariates (including other medications and health care use) (eTable 1 in Supplement 1). We performed model diagnostics (eMethods 3 and eFigures 4 and 5 in Supplement 1). To test whether our results changed under a range of different assumptions, we performed 3 categories of sensitivity analyses: changing the exposure definition, changing the study cohort, and changing the model covariates (Table 1).

As a secondary analysis for the UK cohort, we estimated HRs from a 3-state Cox proportional hazards regression model (1, entry state; 2, received fracture preventive care; and 3, experienced fracture) for switching from one state to another (eFigure 2 in Supplement 1).

In the UK, prescriptions were sometimes missing information on the number of pills to be consumed. We therefore imputed missing values using other information contained in the same prescription, other prescriptions for the same individual, and other prescriptions from the same demographic groups (eMethods 2 in Supplement 1).

We reported any amendments to the original study protocol (eMethods 4 in Supplement 1). Data management and statistical analyses were conducted October 22, 2020, to September 6, 2022, using R, version 4.20 (R Project for Statistical Computing), and SAS Enterprise Guide, version 7.1 (SAS Institute Inc). Statistical significance was defined as a 95% CI excluding 1.

## Results

### Descriptive Statistics

The UK study identified 65 195 people 66 years of age or older (mean [IQR] age, 75 [71-81] years; 65 195 [50.6%] male) with a diagnostic code for eczema, asthma, or COPD who were prescribed 450 mg or more of a prednisolone equivalent dose in 6 months (eFigure 3 in Supplement 1). Of these, 69% had high-intensity oral corticosteroid prescriptions, and 31% had low-intensity prescriptions. For analysis with fracture preventive care medications as the outcome, individuals were followed up for a mean of 0.8 years (total 52 948 person-years), and for analysis with fracture as the outcome for a mean of 3.8 years (total 208 354 person-years) (eTable 2 in Supplement 1). Baseline characteristics were similar between groups of oral corticosteroid prescribing patterns, except for the distribution of inflammatory diseases (high- vs low-intensity prescriptions: eczema 23.5% vs 17.0%; asthma 56.2% vs 61.2%; COPD 55.6% vs 66.2%, respectively) (Table 2).

**Table 2.** Characteristics of the Study Populations at Index Date

Characteristic	UK participants, No. (%)		Ontario participants, No. (%)	
	Oral corticosteroid prescription intensity^a^		Oral corticosteroid prescription intensity^a^	
	High	Low	High	Low
Total No.	44 989	20 206	23 727	4947
Age, mean (IQR), y	75 (71-81)	75 (70-80)	73 (69-79)	73 (69-79)
Male	23 044 (51.2)	9937 (49.2)	14 178 (59.8)	2893 (58.5)
Female	21 945 (48.8)	10 269 (50.8)	9549 (40.2)	2054 (41.5)
Deprivation quintile^b^				
5 (Most deprived)	8606 (19.1)	4428 (21.9)	4970 (20.9)	1169 (23.6)
4	11 584 (25.7)	5437 (26.9)	5185 (21.9)	1087 (22.0)
3	9444 (21.0)	4012 (19.9)	4786 (20.2)	1006 (20.3)
2	7246 (16.1)	2907 (14.4)	4455 (18.8)	917 (18.5)
1 (Least deprived)	5051 (11.2)	1890 (9.4)	4262 (18.0)	754 (15.2)
Missing	3058 (6.8)	1532 (7.6)	69 (0.3)	14 (0.3)
Eczema^c^	10 579 (23.5)	3436 (17.0)	6451 (27.2)	946 (19.1)
Asthma^c^	25 306 (56.2)	12 361 (61.2)	4336 (18.3)	1378 (27.9)
COPD^c^	25 030 (55.6)	13 371 (66.2)	9730 (41.0)	3125 (63.2)
Rheumatoid arthritis	1203 (2.7)	424 (2.1)	790 (3.3)	145 (2.9)

Abbreviation: COPD, chronic obstructive pulmonary disease.

^a^ Low-intensity prescription defined as 90 or more days to cross the risk threshold of the cumulative prednisolone equivalent dose of 450 mg; high-intensity prescription, less than 90 days to cross the risk threshold.

^b^ Quintiles of the Carstairs index in the UK cohort and income quintiles in the Ontario cohort; 1 indicates, least deprived and highest income; 5, most deprived and lowest income.

^c^ Presence of a disease code before the index date; an individual can have multiple diseases.

The study in Ontario identified 28 674 people 66 years of age or older (mean [IQR] age, 73 [69-79] years; 17 071 [59.5%] male), with eczema, asthma, or COPD who were prescribed 450 mg or more of a prednisolone equivalent dose in 6 months. Of these, 82.7% had high-intensity oral corticosteroid prescriptions, whereas 17.3% had low-intensity prescriptions. For analysis with fracture preventive care medications as the outcome, individuals were followed up for a mean of 0.9 years (total 25 600 person-years), and for analysis with fracture as the outcome, for a mean of 5.0 years (total 142 607 person-years) (eTable 2 in Supplement 1). Baseline characteristics were similar between oral corticosteroid prescribing-pattern groups, except for the distribution of inflammatory diseases within 5 years of the index date (high- vs low-intensity prescriptions: eczema, 27.2% vs 19.1%; asthma, 18.3% vs 27.9%; COPD, 41.0% vs. 63.2%, respectively) (Table 2).

### Oral Corticosteroid Prescription Patterns and Fracture Preventive Care

In the UK 1 year after the index date, 8.9% of people who had reached the risk threshold of a 450-mg prednisolone equivalent dose had received fracture preventive care medication: 10.7% with high-intensity oral corticosteroid prescriptions vs 4.8% with low-intensity prescriptions (crude rates, 134 vs 57 per 1000 person-years; crude HR, 2.34; 95% CI, 2.19-2.51; adjusted HR, 2.13; 95% CI, 1.99-2.29) (Table 3). Estimates were similar for the fracture preventive care medication or DEXA scan outcome and lower for the calcium and vitamin D outcome. We saw no evidence for an association between oral corticosteroid prescribing pattern and our negative control outcomes.

**Table 3.** Hazard Ratios for Different Fracture Preventive Care Outcomes, With Follow-Up Maximum of 1 Year, and for Major Osteoporotic Fracture, With Follow-Up Time Not Limited

Cohort	Oral corticosteroid prescription intensity^a^	HR (95% CI)^b^	Person-years	Event	Rate^c^
**Fracture preventive care drugs** ^d^					
UK	Low	1.00 (1.00-1.00)	17 150	971	57
UK	High	2.34 (2.19-2.51)	35 798	4810	134
Ontario	Low	1.00 (1.00-1.00)	4522	219	48
Ontario	High	1.49 (1.29-1.72)	21 078	1534	73
**Fracture preventive care drugs or referral for DEXA scan**					
UK	Low	1.00 (1.00-1.00)	17 105	1061	62
UK	High	2.24 (2.10-2.39)	35 675	5019	141
Ontario	Low	1.00 (1.00-1.00)	4373	501	115
Ontario	High	1.27 (1.15-1.39)	20 302	2959	146
**Calcium and vitamin D**					
UK	Low	1.00 (1.00-1.00)	15 899	2761	174
UK	High	1.46 (1.40-1.53)	33 133	8592	259
**Anxiety drugs** ^e^					
UK	Low	1.00 (1.00-1.00)	15 838	2743	173
UK	High	1.02 (0.98-1.07)	34 737	6208	179
Ontario	Low	1.00 (1.00-1.00)	3817	1181	309
Ontario	High	0.97 (0.91-1.04)	18 207	5494	302
**Epilepsy drugs** ^e^					
UK	Low	1.00 (1.00-1.00)	16 378	1913	117
UK	High	0.97 (0.92-1.02)	36 110	4108	114
**Major osteoporotic fracture (follow-up time not limited)**					
UK	Low	1.00 (1.00-1.00)	74 833	945	13
UK	High	1.07 (0.98-1.15)	169 708	2295	14
Ontario	Low	1.00 (1.00-1.00)	21 775	501	23
Ontario	High	0.87 (0.79-0.96)	120 832	2445	20

Abbrevations: DEXA, dual-energy x-ray absorptiometry; HR, hazard ratio.

^a^ Low intensity: reached risk threshold in 90 to 180 days; high intensity: reached risk threshold within 89 days.

^b^ Hazard ratios (95% CIs) estimated from Cox proportional hazards regression models (with CIs from robust SEs accounting for clustering by general practitioner practice in UK analyses).

^c^ Rate per 1000 person-years.

^d^ Fracture preventive care drugs, including bisphosphonates and other drugs affecting bone metabolism (etidronate, clodronate, bazedoxifene, burosumab, raloxifene, and teriparatide).

^e^ Negative control outcomes.

Analyses by disease subgroup comparing high- vs low-intensity oral corticosteroid prescriptions showed highest HRs for being prescribed fracture preventive care among people with eczema (HR, 3.00; 95% CI, 2.60-3.47) followed by people with asthma (HR, 2.15; 95% CI, 1.96-2.35) and people with COPD (HR, 1.72; 95% CI, 1.57-1.88) (eTable 3 in Supplement 1). Effect estimates were similar in sensitivity analyses with changes to the exposure definition, study cohort composition, and model covariates (Table 4). Using a continuous log-transformed exposure variable (number of days to reach the risk threshold) found an approximate 50% decrease in the hazard of being prescribed fracture preventive care every 10 additional days starting from 0 days (eTable 4 in Supplement 1).

**Table 4.** Hazard Ratios for Being Prescribed Fracture Preventive Care Drugs, From Sensitivity Analyses Comparing High- vs Low-Intensity Oral Corticosteroid Prescriptions

Sensitivity analysis	Hazard ratio (95% CI)^a^	
	UK	Ontario
Main		
Main analysis (maximum follow-up, 1 y)	2.34 (2.19-2.51)	1.49 (1.29-1.72)
Exposure definition change		
Use total length of gaps (0 vs 1-180 d)	3.19 (3.00-3.38)	1.67 (1.52-1.83)
Use total length of gaps (0-89 vs 90-180 d)	2.61 (2.42-2.82)	1.63 (1.39-1.91)
Use No. of gaps (0 vs ≥1)	3.18 (3.00-3.38)	1.67 (1.52-1.83)
Use No. of gaps (0-1 vs ≥2)	2.34 (2.17-2.52)	1.15 (0.97-1.36)
Use time to risk threshold (0 vs 1-180 d)	2.56 (2.38-2.76)	1.49 (1.36-1.65)
Cohort definition change		
Impute more missing values of oral corticosteroid prescriptions	2.50 (2.34-2.67)	
Include all follow-up time	1.49 (1.43-1.55)	
Include people of all ages	2.21 (2.09-2.35)	
Model covariate changes		
Include age, sex, deprivation, comorbidities^b^	2.13 (1.99-2.29)	1.47 (1.27-1.70)
Include age, sex, deprivation	2.36 (2.20-2.53)	
Include age, sex, deprivation, comorbidities, and other^c^		1.37 (1.18-1.59)

^a^ Hazard ratio and 95% CIs estimated from Cox proportional hazards regression models (with CIs from robust SEs accounting for clustering by general practitioner practice in UK analyses).

^b^ Comorbidities: eczema, asthma, rheumatoid arthritis, and chronic obstructive pulmonary disease.

^c^ Other medication: inhaled corticosteroids, injected corticosteroids, topical corticosteroids, other corticosteroids, ever received oral corticosteroids more than 1 year before index date, other drugs affecting fracture risk; health care use: urban or rural home address, number of physician visits in past year (1-12, 13-21, or ≥22), number of hospital admissions (0 or ≥1), number of physicians prescribing oral corticosteroid (1 or ≥2).

In the Ontario study, 1 year after the index date, 6.1% of people who had reached the risk threshold of a 450-mg prednisolone equivalent dose had received fracture preventive care: 6.4% with high-intensity oral corticosteroid prescriptions, and 4.4% with low-intensity prescriptions (crude rates, 73 vs 48 per 1000 person-years, respectively; crude HR, 1.49; 95% CI, 1.29-1.72; adjusted HR, 1.47; 95% CI, 1.27-1.70) (Table 3). Estimates were lower for fracture preventive care medication or DEXA scan outcome. Analyses by disease subgroups comparing people with high- vs low-intensity oral corticosteroid prescriptions found the highest HRs for being prescribed fracture preventive care in people with COPD (HR, 1.58; 95% CI, 1.30-1.91) followed by people with asthma (HR, 1.42; 95% CI, 1.07-1.88) but no substantially increased risk for people with eczema (HR, 1.15; 95% CI, 0.89-1.50) (eTable 3 in Supplement 1). Effect estimates were similar in sensitivity analyses with changes to the exposure definition, study cohort composition, and model covariates.

### Oral Corticosteroid Use Patterns and Fracture

By the end of the UK study period, 5.1% of people who had reached the risk threshold with high-intensity oral corticosteroid prescriptions had experienced a (major osteoporotic) fracture, vs 4.7% with low-intensity prescriptions (crude rates, 14 vs 13 per 1000 person-years; crude HR, 1.07; 95% CI, 0.98-1.15; adjusted HR, 1.12; 95% CI, 1.03-1.21) (Table 3). Effect estimates were similar in sensitivity analyses (eTable 5 in Supplement 1).

By the end of the Ontario study period, 10.3% of people who had reached the risk threshold with high-intensity oral corticosteroid prescriptions had experienced a (major osteoporotic) fracture, vs 10.1% with low-intensity prescriptions (crude rates, 20 vs 23 per 1000 person-years; crude HR, 0.87; 95% CI, 0.79-0.96; adjusted HR, 0.91; 95% CI, 0.73-1.12) (Table 3). Effect estimates were similar in sensitivity analyses (eTable 5 in Supplement 1).

### Results From the 3-State Model

In the UK study, people with high- vs low-intensity oral corticosteroid prescriptions had a higher hazard of moving from the entry state to the fracture preventive care state (HR, 1.53; 95% CI, 1.47-1.60), a somewhat higher hazard for moving from the entry state directly to the fracture state (HR, 1.07; 95% CI, 0.99-1.17), and a slightly lower hazard for moving from the fracture preventive care state to the fracture state (HR, 0.89; 95% CI, 0.75-1.05) albeit with CIs overlapping the null.

## Discussion

In this cohort study conducted in 2 separate populations in the UK and Ontario, Canada, among older people with eczema, asthma, or COPD who received oral corticosteroid prescriptions with a 450-mg prednisolone equivalent dose or higher in 6 months, individuals with a high-intensity prescription pattern were more likely than individuals with a low-intensity pattern to receive fracture preventive care. Except in subgroups of people with eczema, these findings were consistent in parallel cohorts from the UK and Ontario and in analyses that included DEXA scans in the definition of fracture preventive care. People with eczema in the UK study showed the largest increase in rate of fracture preventive care prescribing, but there was no increase in rate in this subgroup in the Ontario study. The UK study found no increase in the rate of fractures among people with low-intensity oral corticosteroid prescriptions, and the Ontario study found a small increase.

While previous studies have explored the association of oral corticosteroid prescribing patterns with the risk of fracture^22^ and fracture preventive care,^23^ we found no studies exploring the association of oral corticosteroid prescribing patterns independent of cumulative dose. Our study conducted that investigation by including only people crossing a risk threshold of a 450-mg prednisolone equivalent dose. The low overall percentage of people in the UK study 1 year after the index date who received fracture preventive care (8.9%) is consistent with previous studies showing low adherence to fracture prevention guidelines for glucocorticoid-induced osteoporosis more generally.^13,14^ In the present study, we identified a population with particularly low rates of fracture preventive care.

Some electronic medical software may provide warnings when high-dose individual oral corticosteroid prescriptions are issued; however, these systems are unlikely to incorporate information on cumulative dose from multiple prescriptions over time.^24^ We found no publicly available information to confirm this assumption. Implementing clinical decision support systems that account for cumulative dose could improve care for people prescribed high-dose oral corticosteroid intermittently; such strategies warrant evaluation. Although fracture risk was similar for people prescribed oral corticosteroids in high- and low-intensity patterns in this study despite differences in fracture preventive care, our study was not designed to assess the efficacy of fracture prevention. There is substantial literature supporting the efficacy of fracture prevention in this population.^8,9,10^

In addition to being important for clinicians practicing family medicine, internal medicine, and respirology, our findings may be particularly important for dermatologists and others treating people with eczema. Oral corticosteroids are not generally recommended for eczema,^25^ but they are still commonly prescribed; in a recent trial for a new biologic to treat eczema, roughly a third of participants reported having used oral corticosteroids.^26^ Therefore, dermatologists and other clinicians caring for people with eczema should minimize oral corticosteroid prescribing and be aware that patients with eczema commonly reach high cumulative oral corticosteroid doses, know the patient’s fracture risk, and consider prescribing fracture prevention or raise the issue with the patient’s primary care team.

In the UK, most people with eczema, asthma, or COPD are managed in primary care. Our UK findings may not apply to people with severe eczema, asthma, or COPD treated in secondary care. Our Ontario cohort includes ambulatory prescriptions from all physicians, including secondary and tertiary care. Different results observed in the present study between the UK and Ontario for subgroups of people with eczema may be due to differences in fracture preventive care prescribing between primary and secondary or tertiary care. For example, there may be greater attention to a patient’s longitudinal eczema treatment, including cumulative oral corticosteroid prescribing, for people with more severe eczema treated in secondary or tertiary care than in primary care, potentially explaining the larger effect estimate found in the UK. Further research could investigate fracture preventive care prescribing for people with skin disease in different countries and health care settings.

### Strengths and Limitations

This study has strengths. Prescribing patterns are analytically challenging due to the need for complex exposure definitions. We conducted multiple sensitivity analyses using varied exposure definitions, and effect estimates were generally similar. We used large, representative population-based databases from 2 countries that offer free access to health care. Similar main analysis results from UK and Canadian data, and broadly consistent results across multiple sensitivity analyses, lend credence to the results.

This study has several limitations. We did not have data on whether medications were taken as prescribed. In the UK, we had only data on whether the prescription was written, and in Ontario, on whether it was filled. There may be other unmeasured confounders, such as frailty, that may explain the association between oral corticosteroid prescribing patterns and receipt of fracture preventive care or fractures. Results from adjusted analyses showed somewhat attenuated HRs. A possible explanation is that some inflammatory diseases of interest may be associated with increased fracture risk independent of oral corticosteroids.^27^ Eczema, asthma, and COPD are treated with topical and inhaled corticosteroids, respectively, but it is controversial whether they are associated with clinically meaningful fracture risk.^28,29^ Null effects observed for all negative control outcomes suggest that there were no major sources of bias.

## Conclusions

In this cohort study conducted in the UK and Ontario, Canada, older adults prescribed high cumulative oral corticosteroid doses gradually or intermittently across multiple prescriptions were approximately half as likely to receive guideline-indicated fracture preventive care compared with older adults receiving similar oral corticosteroid doses in 1 prescription or within a short period of time. These findings suggest missed opportunities to initiate fracture prevention for older people prescribed oral corticosteroids. Clinicians, including dermatologists, respirologists, general practitioners, and internists, should be aware of recent cumulative oral corticosteroid dose, regardless of the prescribing pattern, and initiate fracture preventive care if indicated.

## Notes

Supplement 1. **eMethods 1.** Details on Data Sources **eMethods 2.** OCS Data Cleaning **eMethods 3.** Model Diagnostics **eMethods 4.** Amendments to Study Protocol **eTable 1**. Characteristics of the Ontario Study Population at Index Date **eTable 2.** Total and Average Follow-Up Time (Years) **eTable 3.** Hazard Ratios for Fracture Preventive Care Drugs by Disease Subgroup (Follow-Up Time: Max. 1 Year) **eTable 4.** Hazard Ratios for Fracture Preventive Care Comparing High Intensity to Low Intensity OCS Use With Log-10 Transformed Number of Days to Reach Risk Threshold as the Exposure Variable (Max. 1 Year Follow-Up) **eTable 5.** Hazard Ratios From Sensitivity Analyses Comparing High Intensity to Low Intensity OCS Use for the Major Osteoporotic Fracture Outcome. Outcome: Major Osteoporotic Fracture **eTable 6.** Equivalent Doses of Oral Corticosteroids **eFigure 1.** Example Demonstrating Exposure Definition (Not Real Data) **eFigure 2.** Diagram of States and Possible Directions of Movement Between States **eFigure 3.** Study Flow Diagrams **eFigure 4.** Survival Curves by Exposure **eFigure 5.** Plots of Scaled Schoenfeld Residuals **eAppendix.** RECORD-PE checklist

Supplement 2.
Data Sharing Statement

## References

[1] van Staa TP, Leufkens HGM, Abenhaim L, Zhang B, Cooper C. Oral corticosteroids and fracture risk: relationship to daily and cumulative doses. Rheumatology (Oxford). 2000;39(12):1383-1389. doi:10.1093/rheumatology/39.12.1383 11136882

[2] Lim LS, Hoeksema LJ, Sherin K; ACPM Prevention Practice Committee. Screening for osteoporosis in the adult U.S. population: ACPM position statement on preventive practice. Am J Prev Med. 2009;36(4):366-375. doi:10.1016/j.amepre.2009.01.013 19285200

[3] Boling EP. Secondary osteoporosis: underlying disease and the risk for glucocorticoid-induced osteoporosis. Clin Ther. 2004;26(1):1-14. doi:10.1016/S0149-2918(04)90001-X 14996513

[4] Burge R, Dawson-Hughes B, Solomon DH, Wong JB, King A, Tosteson A. Incidence and economic burden of osteoporosis-related fractures in the United States, 2005-2025. J Bone Miner Res. 2007;22(3):465-475. doi:10.1359/jbmr.061113 17144789

[5] Compston J. Glucocorticoid-induced osteoporosis: an update. Endocrine. 2018;61(1):7-16. doi:10.1007/s12020-018-1588-2 29691807 PMC5997116

[6] Kanis JA, Oden A, Johansson H, Borgström F, Ström O, McCloskey E. FRAX and its applications to clinical practice. Bone. 2009;44(5):734-743. doi:10.1016/j.bone.2009.01.373 19195497

[7] FRAX. Fracture risk assessment tool. Accessed June 24, 2023. https://frax.shef.ac.uk/FRAX/

[8] National Institute for Health and Care Excellence. Clinical guideline [CG146]: osteoporosis: assessing the risk of fragility fracture. Published August 8, 2012; updated February 7, 2017. Accessed June 25, 2023. https://www.nice.org.uk/guidance/cg146

[9] Papaioannou A, Morin S, Cheung AM, ; Scientific Advisory Council of Osteoporosis Canada. 2010 clinical practice guidelines for the diagnosis and management of osteoporosis in Canada: summary. CMAJ. 2010;182(17):1864-1873. doi:10.1503/cmaj.100771 20940232 PMC2988535

[10] American College of Rheumatology. American College of Rheumatology guideline for the prevention and treatment of glucocorticoid-induced osteoporosis guideline. 2022. Accessed May 17, 2023. https://rheumatology.org/glucocorticoid-induced-osteoporosis-guideline 10.1002/art.42646 37845798

[11] National Institute for Health and Care Excellence. Rheumatoid arthritis in adults: management. Last updated October 12, 2020. Accessed June 25, 2023. https://www.nice.org.uk/guidance/ng100/chapter/Recommendations

[12] Alexander T, Maxim E, Cardwell LA, Chawla A, Feldman SR. Prescriptions for atopic dermatitis: oral corticosteroids remain commonplace. J Dermatolog Treat. 2018;29(3):238-240. doi:10.1080/09546634.2017.1365112 28789575

[13] Bénard-Laribière A, Pariente A, Pambrun E, Bégaud B, Fardet L, Noize P. Prevalence and prescription patterns of oral glucocorticoids in adults: a retrospective cross-sectional and cohort analysis in France. BMJ Open. 2017;7(7):e015905. doi:10.1136/bmjopen-2017-015905 28760791 PMC5642779

[14] Liao K-M, Chiu K-L, Chen C-Y. Prescription patterns in patients with chronic obstructive pulmonary disease and osteoporosis. Int J Chron Obstruct Pulmon Dis. 2021;16:761-769. doi:10.2147/COPD.S289799 33790552 PMC8007556

[15] Nazrun AS, Tzar MN, Mokhtar SA, Mohamed IN. A systematic review of the outcomes of osteoporotic fracture patients after hospital discharge: morbidity, subsequent fractures, and mortality. Ther Clin Risk Manag. 2014;10:937-948. 25429224 10.2147/TCRM.S72456 PMC4242696

[16] Herrett E, Gallagher AM, Bhaskaran K, . Data resource profile: clinical practice research datalink (CPRD). Int J Epidemiol. 2015;44(3):827-836. doi:10.1093/ije/dyv098 26050254 PMC4521131

[17] Wheeler B. Carstairs Index 2011 for lower-layer super output areas. Published 2019. Accessed June 25, 2023. https://reshare.ukdataservice.ac.uk/851497/

[18] Gershon AS, Wang C, Guan J, Vasilevska-Ristovska J, Cicutto L, To T. Identifying patients with physician-diagnosed asthma in health administrative databases. Can Respir J. 2009;16(6):183-188. doi:10.1155/2009/963098 20011725 PMC2807792

[19] Gershon AS, Wang C, Guan J, Vasilevska-Ristovska J, Cicutto L, To T. Identifying individuals with physician diagnosed COPD in health administrative databases. COPD. 2009;6(5):388-394. doi:10.1080/15412550903140865 19863368

[20] O’Donnell S; Canadian Chronic Disease Surveillance System (CCDSS) Osteoporosis Working Group. Use of administrative data for national surveillance of osteoporosis and related fractures in Canada: results from a feasibility study. Arch Osteoporos. 2013;8:143. doi:10.1007/s11657-013-0143-2 23740086 PMC5096934

[21] Arnold BF, Ercumen A. Negative control outcomes: a tool to detect bias in randomized trials. JAMA. 2016;316(24):2597-2598. doi:10.1001/jama.2016.17700 28027378 PMC5428075

[22] De Vries F, Bracke M, Leufkens HGM, Lammers JW, Cooper C, Van Staa TP. Fracture risk with intermittent high-dose oral glucocorticoid therapy. Arthritis Rheum. 2007;56(1):208-214. doi:10.1002/art.22294 17195223

[23] Liu RH, Albrecht J, Werth VP. Cross-sectional study of bisphosphonate use in dermatology patients receiving long-term oral corticosteroid therapy. Arch Dermatol. 2006;142(1):37-41. doi:10.1001/archderm.142.1.37 16415384

[24] Ardens Health Informatics. NPSA Steroid Alerts. 2022. Accessed June 25, 2023. https://support-ew.ardens.org.uk/support/solutions/articles/31000158989-npsa-steroid-alerts

[25] Drucker AM, Eyerich K, de Bruin-Weller MS, . Use of systemic corticosteroids for atopic dermatitis: International Eczema Council consensus statement. Br J Dermatol. 2018;178(3):768-775. doi:10.1111/bjd.15928 28865094 PMC5901393

[26] Simpson EL, Gooderham M, Wollenberg A, ; ADhere Investigators. Efficacy and safety of lebrikizumab in combination with topical corticosteroids in adolescents and adults with moderate-to-severe atopic dermatitis: a randomized clinical trial (ADhere). JAMA Dermatol. 2023;159(2):182-191. doi:10.1001/jamadermatol.2022.5534 36630140 PMC9857439

[27] Matthewman J, Mansfield KE, Prieto-Alhambra D, . Atopic eczema–associated fracture risk and oral corticosteroids: a population-based cohort study. *J Allergy Clin Immunol Pract*. 2022;10(1):257-266. 10.1016/j.jaip.2021.09.026 PMC7612204 34571200

[28] Loke YK, Gilbert D, Thavarajah M, Blanco P, Wilson AM. Bone mineral density and fracture risk with long-term use of inhaled corticosteroids in patients with asthma: systematic review and meta-analysis. BMJ Open. 2015;5(11):e008554. doi:10.1136/bmjopen-2015-008554 26603243 PMC4663435

[29] Egeberg A, Schwarz P, Harsløf T, . Association of potent and very potent topical corticosteroids and the risk of osteoporosis and major osteoporotic fractures. *JAMA Dermatol*. 2021;157(3):275-282. doi:10.1001/jamadermatol.2020.4968 PMC7970335 33471030

