# Supplementary material for: Association of Different Prescribing Patterns for Oral Corticosteroids With Fracture Preventive Care Among Older Adults in the UK and Ontario
Source: JAMA Dermatol. 2023 Aug 9;159(9):961–9. doi: 10.1001/jamadermatol.2023.2495 (PMC10413212; doi:10.1001/jamadermatol.2023.2495)
Supplement: Supplement 1. — eMethods 1. Details on Data Sources eMethods 2. OCS Data Cleaning eMethods 3. Model Diagnostics eMethods 4. Amendments to Study Protocol eTable 1. Characteristics of the Ontario Study Population at Index Date eTable 2. Total and Average Follow-Up Time (Years) eTable 3. Hazard Ratios for Fracture Preventive Care Drugs by Disease Subgroup (Follow-Up Time: Max. 1 Year) eTable 4. Hazard Ratios for Fracture Preventive Care Comparing High Intensity to Low Intensity OCS Use With Log-10 Transformed Number of Days to Reach Risk Threshold as the Exposure Variable (Max. 1 Year Follow-Up) eTable 5. Hazard Ratios From Sensitivity Analyses Comparing High Intensity to Low Intensity OCS Use for the Major Osteoporotic Fracture Outcome. Outcome: Major Osteoporotic Fracture eTable 6. Equivalent Doses of Oral Corticosteroids eFigure 1. Example Demonstrating Exposure Definition (Not Real Data) eFigure 2. Diagram of States and Possible Directions of Movement Between States eFigure 3. Study Flow Diagrams eFigure 4. Survival Curves by Exposure eFigure 5. Plots of Scaled Schoenfeld Residuals eAppendix. RECORD-PE checklist [file jamadermatol-e232495-s001.pdf]

Supplementary Online Content

Matthewman J, Tadrous M, Mansfield KE, et al. Association of different prescribing patterns for oral corticosteroids with fracture preventive care among older adults in the UK and Ontario. Published online August 9, 2023. *JAMA Dermatol*. doi:10.1001/jamadermatol.2023.2495

eMethods 1. Details on Data Sources
eMethods 2. OCS Prescription Data Cleaning
eMethods 3. Model Diagnostics
eMethods 4. Amendments to Study Protocol
eTable 1. Characteristics of the Ontario Study Population at Index Date
eTable 2. Total and Average Follow-Up Time (Years)
eTable 3. Hazard Ratios for Fracture Preventive Care Drugs by Disease Subgroup (Follow-Up Time: Max. 1 Year)
eTable 4. Hazard Ratios for Fracture Preventive Care Comparing High Intensity to Low Intensity OCS Use With Log-10 Transformed Number of Days to Reach Risk Threshold as the Exposure Variable (Max. 1 Year Follow-Up)
eTable 5. Hazard ratios From Sensitivity Analyses Comparing High Intensity to Low Intensity OCS Use for the Major Osteoporotic Fracture Outcome. Outcome: Major Osteoporotic Fracture
eTable 6. Equivalent Doses of Oral Corticosteroids
eFigure 1. Example Demonstrating Exposure Definition (Not Real Data)
eFigure 2. Diagram of States and Possible Directions of Movement Between States
eFigure 3. Study Flow Diagrams
eFigure 4. Survival Curves by Exposure
eFigure 5. Plots of Scaled Schoenfeld Residuals
eAppendix. RECORD-PE checklist
eReferences.

This supplementary material has been provided by the authors to give readers additional information about their work.

© 2023 Matthewman J et al. *JAMA Dermatology*.

A.1 eMethods

eMethods 1: Details on Data Sources

UK:

- Linked data from the Office for national statistics (ONS): Death registration.
- Small area level data: Patient postcode and practice postcode linked Carstairs Index using 2011 census data. The Carstairs index is an index of material deprivation at the small area level, based on four measures from the UK census.[29]
- Clinical practice research datalink (CPRD): All other variables.

Ontario:

- Registered Persons Database (RPDB): Date of birth and death, sex, postal code (updated annually); use Canadian census data to allow neighbourhood-level income estimates.[30, 31]
- Canadian Institutes of Health Information Discharge Abstract Database (CIHI-DAD): Demographic and clinical information about all hospital admissions and discharges, including transfers and deaths using standard diagnosis (ICD-9/ICD-10-CA) and procedure/intervention codes (CCP/CCI). In a hospital medical record re-abstraction study (14,500 hospital discharges, 18 Ontario hospitals), median agreement between the original and re-abstracted records for the 50 most common diagnoses was 81%.[32]
- National Ambulatory Care Reporting System (NACRS): All hospital- and community-based ambulatory care including emergency department (ED) visits; ED discharge diagnoses are mapped to ICD-10 codes.
- Ontario Health Insurance Plan (OHIP): Physicians submit claims for each service provided, with diagnostic codes based on ICD criteria. Outpatient visits are complete and reliable.[33]
- Ontario Drug Benefit Database (ODB): Prescription medication data is collected for Ontarians ≥65 years old with an error rate of <1%.[34, 35]
- ICES Physician Database (IPDB): Contains data on physician specialty.
- Ontario Cancer Registry (OCR): Computerized database on all people in Ontario diagnosed with cancer since 1965, maintained by Cancer Care Ontario. 95% of all cancers captured.[36]

We obtained individuals’ age, sex, and home location at the time of the index date from RPDB. We identified eczema, asthma, COPD, rheumatoid arthritis, and dementia during a 5-year look back requiring at least two physician visits with the diagnosis in the OHIP database. We identified medications that may increase or decrease fracture risk, including other types of corticosteroids (inhaled/nasal, injectable, topical and other) used in the year prior to index date from ODB. We identified healthcare utilisation during the year prior to the index date in OHIP and DAD databases, including the number of physician visits and hospital visits. We established the specialty of the physician, or physicians, prescribing any OCS that contributed to crossing the risk threshold from ODB and ICES physician database. As a measure of socio-economic status, we used quintiles based on neighbourhood income using the Statistics Canada algorithm.

© 2023 Matthewman J et al. *JAMA Dermatology*.

eMethods 2. OCS Prescription Data Cleaning

UK: We identified prescriptions for oral corticosteroids from primary care therapy data including information on the substance, start date, quantity and daily dose. We identified substance classes, and calculated the prednisolone equivalent dose for each prescription using conversion factors from eTable 6.[37] We then identified the total prednisolone equivalent dose and duration for each prescription. We used a "Hot Deck" approach to imputation.[38] We imputed missing values for quantity by taking the median of the patient's prescription quantity and if this was not available, the median of all prescription quantities. We imputed missing values (and 0 values as a sensitivity analysis) for daily dose by taking the median value from (in order of most preferred to least proffered) 1. the same patient, same dose and quantity, 2. the same patient, same dose and quantity group (quantity above and below 42), 3. the same patient with same dose 4. people from the same age group, sex, dose and quantity 5. people from the same age group, sex, dose and quantity group.

Ontario: Prescription claims and drug identification numbers from the Ontario Drug Benefit Plan (ODB) database were used to calculate prednisolone equivalent dose for each prescription (without imputation of missing values).

eMethods 3. Model Diagnostics

We checked the proportional hazards assumption for Cox models by plotting survival curves and Schoenfeld residual plots for the fracture and fracture preventive care outcomes for the exposure (high-intensity vs low-intensity OCS use). The Schoenfeld Individual Test p values was significant for the fracture preventive care outcome, with the curve gradually approaching 0, suggesting that the prescription pattern (high intensity vs low intensity) is most strongly associated with the outcome at the beginning of follow-up, dropping to almost no associated by the end of the year. Given we limited the follow-up time to one year, we concluded it is reasonable to use Cox proportional hazards models, although there may be some violation of the proportional hazards assumption. eFigure 4 eFigure 5

eMethods 4. Amendments to Study Protocol

We changed two aspects of the study design after the original protocol was submitted, as was approved by the CPRD's Independent Scientific Advisory Committee (ISAC Protocol Number 22_002190).

Firstly, we changed the study population from people with eczema, as was originally proposed, to people with eczema, asthma or COPD. This was done to better represent the population of people receiving oral corticosteroids in gradual or intermittent patterns, and to make findings useful for researchers and guideline authors focusing on specific inflammatory diseases.

Secondly, we changed our exposure definition. Our originally proposed exposure definition was based on measuring the proportion of time a participant was prescribed OCSs in consecutive 90-day windows after the index date. Through visualisation of a sample of participants prescription timelines, and implementation of negative control outcomes, we recognised that this exposure definition was likely prone to time-dependent bias, and we therefore changed the exposure definition to using only information occurring before the index date.

© 2023 Matthewman J et al. *JAMA Dermatology*.

A.2 eTables

eTable 1. Characteristics of the Ontario Study Population at Index Date

Characteristic	Level	high intensity, N=23,727	low intensity, N=4,947
Age	Age	73 (69-79)	73 (69-79)
Sex	Male	14,178 (59.8%)	2,893 (58.5%)
Income	Lowest	4,970 (20.9%)	1,169 (23.6%)
	Next to lowest	5,185 (21.9%)	1,087 (22.0%)
	Middle	4,786 (20.2%)	1,006 (20.3%)
	Next to highest	4,455 (18.8%)	917 (18.5%)
	Highest	4,262 (18.0%)	754 (15.2%)
	Missing	69 (0.3%)	14 (0.3%)
Eczema	Eczema	6,451 (27.2%)	946 (19.1%)
Asthma	Asthma	4,336 (18.3%)	1,378 (27.9%)
COPD	COPD	9,730 (41.0%)	3,125 (63.2%)
Rheumatoid arthritis	Rheumatoid arthritis	790 (3.3%)	145 (2.9%)
Rurality	Urban	19,167 (80.8%)	4,028 (81.4%)
	Rural	*4533 - 4537	*914 - 918
	Missing	*23 - 27	*1 - 5
Physician visits in the year prior to index date	0-12 physician visits	8,774 (37.0%)	1,539 (31.1%)
	13-21 physician visits	7,392 (31.2%)	1,617 (32.7%)
	22+ physician visits	7,561 (31.9%)	1,791 (36.2%)
Hospitalisations in the year prior to index date	hospital admission=0	17,296 (72.9%)	3,287 (66.4%)
	hospital admission>=1	6,431 (27.1%)	1,660 (33.6%)
Number of physicians prescribing OCS	Missing	1,089 (4.6%)	97 (2.0%)
	1 physician	14,619 (61.6%)	1,854 (37.5%)
	2+ physician	8,019 (33.8%)	2,996 (60.6%)
Index diagnosis: Eczema	Yes	10,682 (45.0%)	1,088 (22.0%)
Index diagnosis: Asthma	Yes	2,826 (11.9%)	760 (15.4%)
Index diagnosis: COPD	Yes	10,307 (43.4%)	3,116 (63.0%)
Dementia within 5 years prior to index date	Yes	1,446 (6.1%)	263 (5.3%)
Rheumatoid arthritis within 5 years prior to index date	Yes	790 (3.3%)	145 (2.9%)
Inhaled steroid within 1 year prior to index date	Yes	5,985 (25.2%)	1,645 (33.3%)
Injectable steroid within 1 year prior to index date	Yes	962 (4.1%)	215 (4.3%)
Topical steroid within 1 year prior to index date	Yes	9,157 (38.6%)	1,484 (30.0%)
Other medications affecting fracture risk within 1 year prior to index date	Yes	1,513 (6.4%)	370 (7.5%)
Oral corticosteroids within 1 year prior to index date	Yes	6,019 (25.4%)	1,481 (29.9%)
Year of index date	2002-2005	4,119 (17.4%)	501 (10.1%)
	2006-2012	8,094 (34.1%)	1,441 (29.1%)
	2013-2019	11,514 (48.5%)	3,005 (60.7%)

*Values given as ranges to avoid small cells for re-identification purposes

© 2023 Matthewman J et al. *JAMA Dermatology*.

eTable 2: Total and average follow-up time (years)

Cohort	overall[1]	high intensity (0-89 days)[1]	low intensity (90-180 days)[1]	analysis
**FPC drugs**				
UK	52,948 (0.8)	35,798 (0.8)	17,150 (0.8)	Max. 1 year follow-up
UK	208,354 (3.2)	141,294 (3.1)	67,060 (3.3)	Include all follow up time
Ontario	25,600 (0.9)	21,078 (0.9)	4,522 (0.9)	Max. 1 year follow-up
**Major osteoporotic fracture**				
UK	56,106 (0.9)	38,550 (0.9)	17,555 (0.9)	Max. 1 year follow-up
UK	244,541 (3.8)	169,708 (3.8)	74,833 (3.7)	Include all follow up time
Ontario	142,607 (5.0)	120,832 (5.1)	21,775 (4.4)	Include all follow up time
[1] Years: total (average)				

© 2023 Matthewman J et al. *JAMA Dermatology*.

eTable 3: Hazard Ratios For Fracture Preventive Care Drugs by Disease Subgroup (Follow-Up Time: Max. 1 Year)

Cohort	Pattern[1]	HR (95%CI)[2,3]	person-years	event	rate[4]
People with COPD[5]					
UK	low intensity	1.00 (1.00-1.00)	11,266	615	55
UK	high intensity	1.72 (1.57-1.88)	20,112	1,896	94
Ontario	low intensity	1.00 (1.00-1.00)	2,752	260	94
Ontario	high intensity	1.58 (1.30-1.91)	8,643	1,116	129
People with eczema[5]					
UK	low intensity	1.00 (1.00-1.00)	2,848	212	74
UK	high intensity	3.00 (2.60-3.47)	7,910	1,816	230
Ontario	low intensity	1.00 (1.00-1.00)	940	161	171
Ontario	high intensity	1.15 (0.89-1.50)	9,242	1,505	163
People with asthma[5]					
UK	low intensity	1.00 (1.00-1.00)	10,697	576	54
UK	high intensity	2.15 (1.96-2.35)	20,788	2,425	117
Ontario	low intensity	1.00 (1.00-1.00)	694	83	120
Ontario	high intensity	1.42 (1.07-1.88)	2,493	351	141

[1] Oral corticosteroid prescription pattern, either low intensity (reached risk threshold within 90 to 180 days) or high intensity (reached risk threshold within 89 days.

[2] Hazard ratios (95% confidence intervals) estimated from Cox models (with confidence intervals from robust standard errors accounting for clustering by GP practice in UK analyses). The dotted line represents the null (HR=1), the square and error bar the estimated hazard ratio and confidence interval respectively.

[3] Fracture preventive care (FPC) drugs, including bisphosphonates and other drugs affecting bone metabolism (etidronate, clodronate, bazedoxifene, burosumab, raloxifene, teriparatide).

[4] Rate per 1,000 person-years

[5] Individuals with a diagnostic code for the respective inflammatory disease any time before index date (UK)/ within 5 years prior to index date (Ontario). Individuals can have more than one inflammatory disease.

© 2023 Matthewman J et al. *JAMA Dermatology*.

eTable 4. Hazard Ratios for Fracture Preventive Care Comparing High Intensity to Low Intensity OCS Use With Log-10 Transformed Number of Days to Reach Risk Threshold as the Exposure Variable (Max. 1 Year Follow-Up)

	UK		Ontario	
**Outcome**	**HR (95%CI)[1]**	**Adjusted HR (95%CI)[2]**	**HR (95%CI)[1]**	**Adjusted HR (95%CI)[2]**
Fracture preventive care	0.56 (0.54-0.58)	0.60 (0.58-0.63)	0.84 (0.81-0.88)	
Bisphosphonates	0.55 (0.53-0.57)	0.59 (0.57-0.61)	0.78 (0.74-0.83)	0.77 (0.72-0.81)
Calcium & Vitamin D	0.75 (0.72-0.77)	0.78 (0.76-0.81)		
DXA Scans	0.84 (0.74-0.94)	0.87 (0.77-0.99)		
Major osteoporotic fracture	0.96 (0.87-1.06)	0.93 (0.84-1.03)	1.04 (0.94-1.15)	
Anxiety drugs[3]	1.01 (0.98-1.05)	0.99 (0.96-1.02)		
Epilepsy drugs[3]	1.07 (1.03-1.11)	1.06 (1.02-1.10)		
Migraine drugs[3]	0.91 (0.77-1.08)	0.97 (0.81-1.16)		

[1] Crude Hazard ratios (95% confidence intervals) from Cox models estimating the hazard for a 10 day increase in the number of days taken to reach the risk threshold

[2] adjusted for age, sex, deprivation, eczema, asthma, COPD, and rheumatoid arthritis

[3] negative control outcomes

© 2023 Matthewman J et al. *JAMA Dermatology*.

eTable 5. Hazard Ratios From Sensitivity Analyses Comparing High Intensity to Low Intensity OCS Use for the Major Osteoporotic Fracture Outcome. Outcome: Major Osteoporotic Fracture

	HR (95%CI)[1]			
Sensitivity analysis	UK		Ontario	
**Main**				
Main analysis (follow- up not limited)		1.07 (0.98-1.15)		0.87 (0.79-0.96)
**Changed exposure definition**				
Define exposure using total length of gaps (0 vs 1-180)		0.98 (0.91-1.07)		0.95 (0.88-1.02)
Define exposure using total length of gaps (0-89 vs 90-180)		1.07 (0.98-1.17)		0.89 (0.81-0.99)
Define exposure using number of gaps (0 vs 1+)		0.98 (0.91-1.07)		0.95 (0.88-1.02)
Define exposure using number of gaps (0-1 vs 2+)		0.96 (0.89-1.03)		0.85 (0.75-0.97)
Define exposure using time to risk threshold (0 vs 1-180)		0.93 (0.84-1.02)		0.92 (0.85-0.99)
**Changed model covariates**				
+ age, sex, deprivation, comorbidities[2]		1.12 (1.03-1.21)		0.93 (0.85-1.03)
+ age, sex, deprivation		1.09 (1.00-1.19)		
+ age, sex, deprivation, comorbidities, other[3]				0.93 (0.84-1.03)

[1] Hazard ratios (95% confidence intervals) estimated from Cox models (with confidence intervals from robust standard errors accounting for clustering by GP practice in UK analyses). The dotted line represents the null (HR=1), the square and error bar the estimated hazard ratio and confidence interval respectively.

[2] Comorbidities: asthma, rheumatoid arthritis, chronic obstructive pulmonary disease (COPD).

[3] Other medication: inhaled corticosteroids, injected corticosteroids, topical corticosteroids, other corticosteroids, ever received oral corticosteroids more than 1 year before index date, other drugs affecting fracture risk; Healthcare utilisation: urban/rural home address, number of physician visits in past year (1-12/13-21/22+), number of hospital admissions (0/1+), number of physicians prescribing OCS (1/2+).

© 2023 Matthewman J et al. *JAMA Dermatology*.

eTable 6. Equivalent Doses of Oral Corticosteroids

**Drug**	**Dose equivalent to 1mg prednisolone**
Betamethasone	0.15
Betamethasone sodium phosphate	0.15
Deflazacort	1.20
Dexamethasone	0.15
Dexamethasone Sodium Phosphate	0.15
Dexamethasone sodium phosphate	0.15
Hydrocortisone	4.00
Hydrocortisone Acetate	4.00
Hydrocortisone Sodium Phosphate	4.00
Hydrocortisone Sodium Succinate	4.00
Hydrocortisone acetate	4.00
Hydrocortisone sodium phosphate	4.00
Hydrocortisone sodium succinate	4.00
Methylprednisolone	0.80
Methylprednisolone Acetate	0.80
Methylprednisolone acetate	0.80
Methylprednisolone sodium succinate	0.80
Prednisolone	1.00
Prednisolone Sodium Phosphate	1.00
Prednisolone Steaglate	1.00
Prednisolone acetate	1.00
Prednisolone sodium phosphate	1.00
Prednisone	1.00
Triamcinolone Acetonide	0.80
Triamcinolone Hexacetonide	0.80
Triamcinolone acetonide	0.80
Triamcinolone hexacetonide	0.80

© 2023 Matthewman J et al. *JAMA Dermatology*.

A.3 eFigures

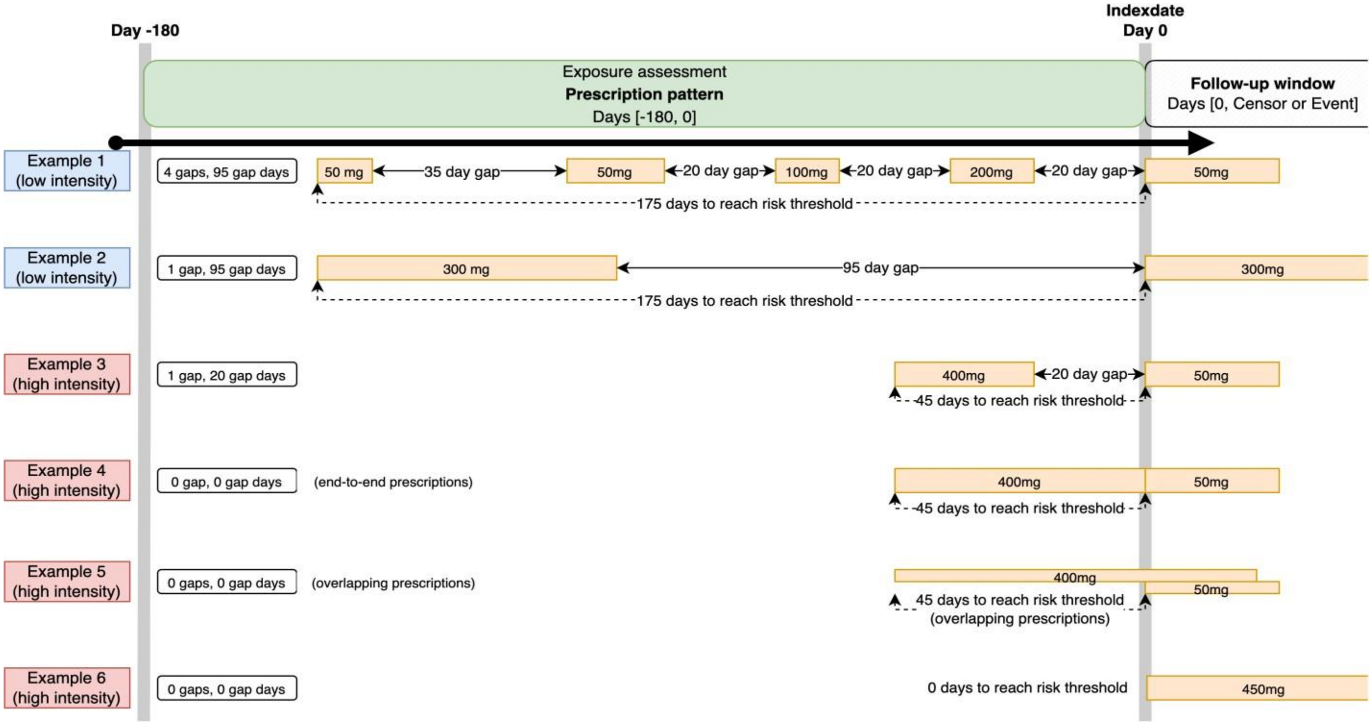

eFigure 1. Example Demonstrating Exposure Definition (Not Real Data)

6 different examples, each showing an individual's oral corticosteroid (OCS) prescriptions with prednisolone equivalent dose (PED) (in orange) in the 180 days (approximately 6 months) leading up to the time of crossing the risk threshold of 450mg PED (index date). We categorised OCS use according to the number of days taken to reach the risk threshold of 450mg PED within 180 days (main analysis), and according to the number, and total length of gaps within that time (sensitivity analyses).

© 2023 Matthewman J et al. *JAMA Dermatology*.

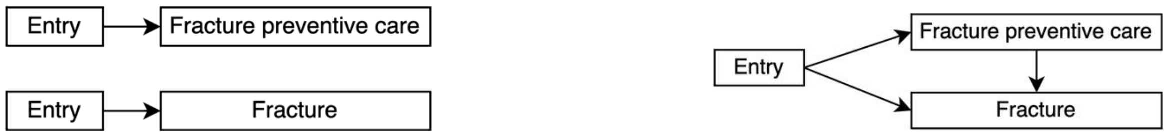

(a) Main analyses (two separate two-state analyses)

(b) Multistate analysis (one three-state analysis)

eFigure 2. Diagram of States and Possible Directions of Movement Between States

Diagrams showing the different analytic approach used for the main analyses (two-state analyses for both the fracture preventive care and fracture outcomes), and the multi-state analysis (three-state analysis combining both outcomes). In the multi-state analysis, people are censored only when they experience a fracture (absorbing state, i.e. cannot switch to another state thereafter), but not when they receive fracture preventive care.

© 2023 Matthewman J et al. *JAMA Dermatology*.

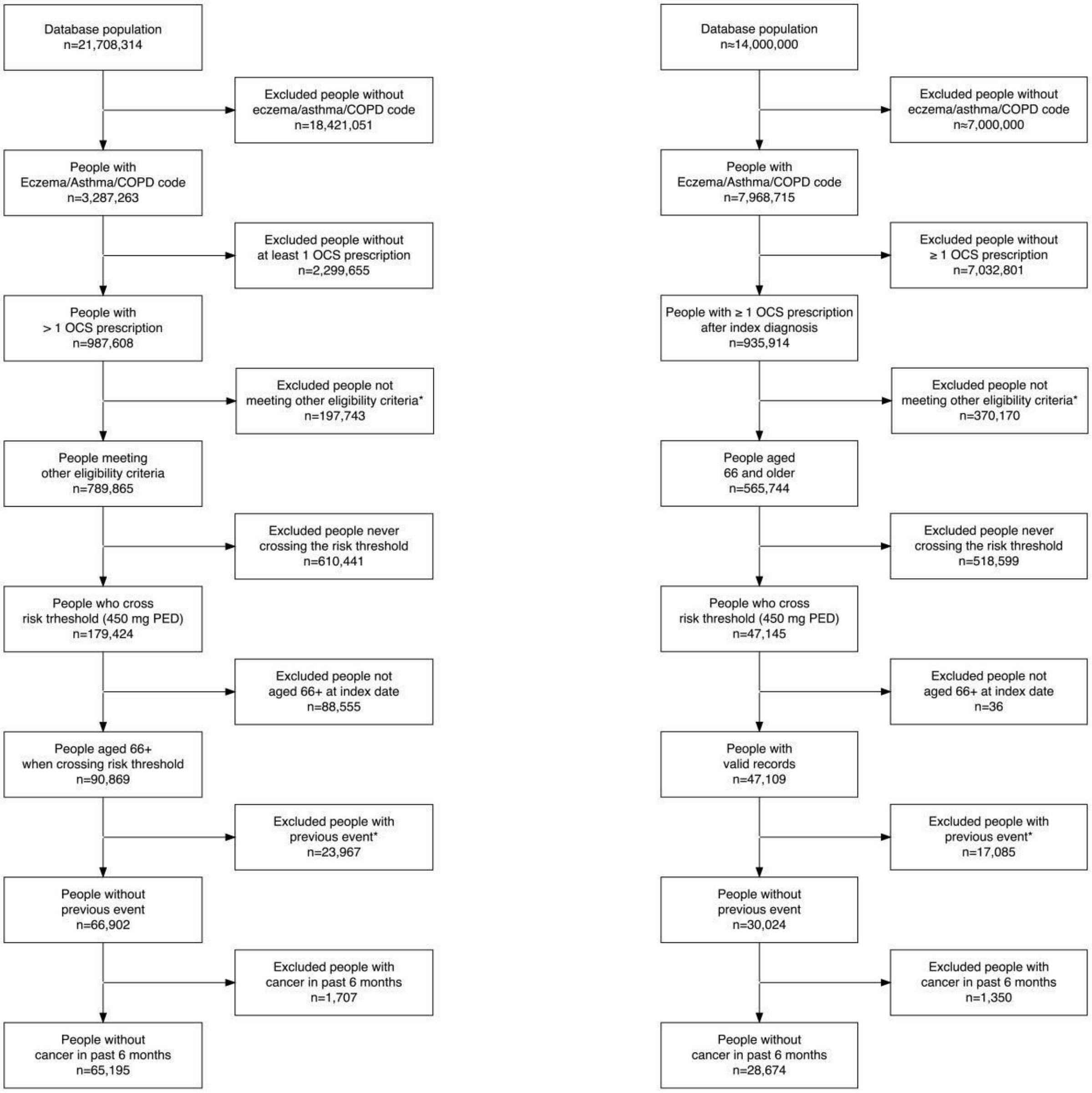

UK Ontario

eFigure 3. Study Flow Diagrams

Number of participants and person-years at each step of the data management process.
UK database population: People ever registered in a CPRD eligible UK general practice from January 2nd 1998 to January 31st 2020 from CPRD.
Ontario database population: administrative health data from April 1st 2002 to September 30th 2020 from ICES data sources.
* Previous event: previous prescription for a fracture preventive care drug or major osteoporotic fracture.
* Other eligibility criteria: at least 18 years old, study start date has passed (UK ≥ January 2, 1998; *Ontario:* ≥ 1st April 2002), practice meets quality standards (UK only), 12 months registration at eligible practice (UK only).

© 2023 Matthewman J et al. *JAMA Dermatology*.

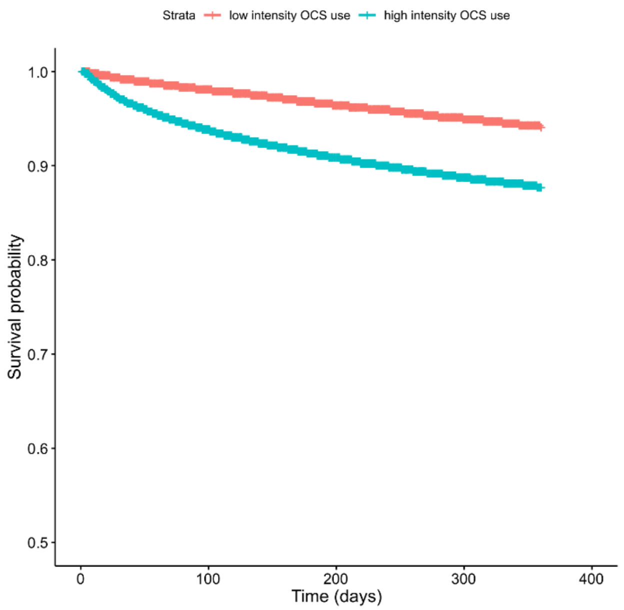

for the fracture preventive care outcome

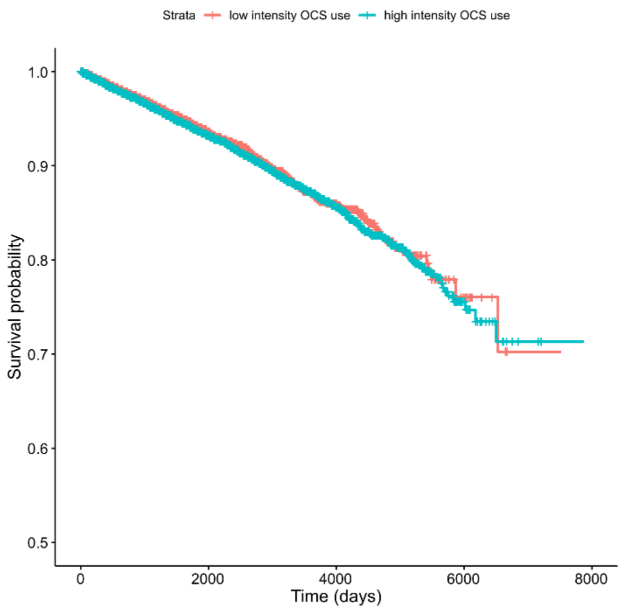

for the fracture outcome

eFigure 4. Survival Curves by Exposure

Survival curves showing the probability of survival (i.e. staying outcome-free) over time for the fracture preventive care (follow-up time limited to one year) and fracture (follow-up time not limited) outcomes.

© 2023 Matthewman J et al. *JAMA Dermatology*.

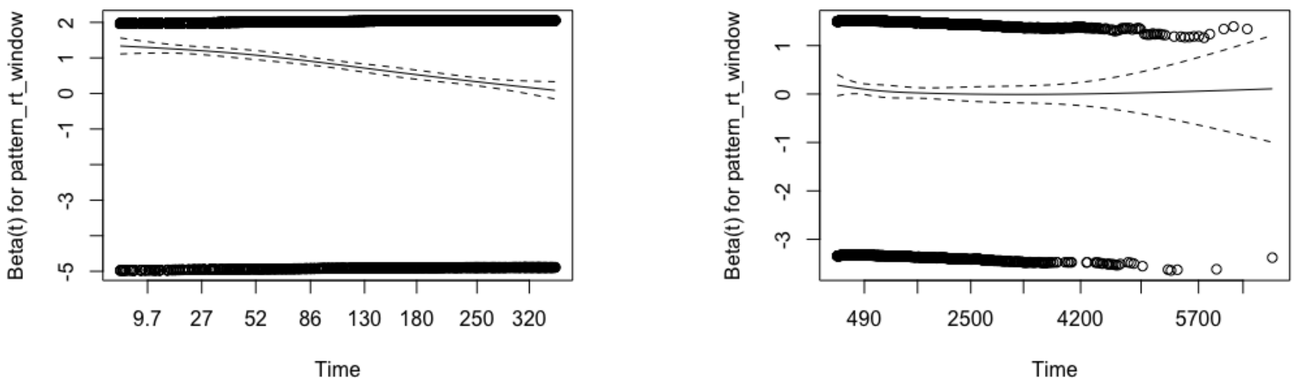

(a) for the fracture preventive care outcome *(b) for the fracture outcome*

eFigure 5. Plots of Scaled Schoenfeld Residuals

Plots of scaled Schoenfeld residuals, along with smoothed curves, to estimate the time dependence of the exposure for the outcome. For the fracture preventive care outcome, the curve gradually approaching 0 suggests that the prescription pattern (high intensity vs low intensity) is most strongly associated with the outcome at the beginning of follow-up, dropping to almost no associated by the end of the year. For the fracture outcome, where follow-up time was not limited, the curve being approximately horizontal and close to 0 suggests proportional hazards.

© 2023 Matthewman J et al. *JAMA Dermatology*.

A.4 eAppendix. RECORD-PE checklist

The RECORD statement for pharmacoepidemiology (RECORD-PE) checklist of items, extended from the STROBE and RECORD statements, which should be reported in non-interventional pharmacoepidemiological studies using routinely collected health data.[39]

Title and abstract

1. Title and abstract

- (STROBE) Indicate the study's design with a commonly used term in the title or the abstract. Abstract
- (STROBE) Provide in the abstract an informative and balanced summary of what was done and what was found. Abstract
- (RECORD) The type of data used should be specified in the title or abstract. When possible, the name of the databases used should be included. Abstract
- (RECORD) If applicable, the geographical region and timeframe within which the study took place should be reported in the title or abstract. Abstract
- (RECORD) If linkage between databases was conducted for the study, this should be clearly stated in the title or abstract. Abstract

Introduction

2. Background rationale

- (STROBE) Explain the scientific background and rationale for the investigation being reported. Background

3. Objectives

- (STROBE) State specific objectives, including any prespecified hypotheses. Background

Methods

4. Study design

- (STROBE) Present key elements of study design early in the paper. Study design and setting
- (RECORD-PE) Include details of the specific study design (and its features) and report the use of multiple designs if used. Study design and setting
- (RECORD-PE) The use of a diagram(s) is recommended to illustrate key aspects of the study design(s), including exposure, washout, lag and observation periods, and covariate definitions as relevant. Figure 1

5. Setting

- (STROBE) Describe the setting, locations, and relevant dates, including periods of recruitment, exposure, follow-up, and data collection. Data sources, Study population

6. Participants

- (STROBE) Cohort study—give the eligibility criteria, and the sources and methods of selection of participants. Describe methods of follow-up. ~~Case-control study—give the eligibility criteria, and the sources and methods of case ascertainment and control selection. Give the rationale for the choice of cases and controls. Cross sectional study—give the eligibility criteria, and the sources and methods of selection of participants.~~ Exposures, outcomes, and covariates Statistical analyses

© 2023 Matthewman J et al. *JAMA Dermatology*.

- ~~(STROBE) Cohort study—for matched studies, give matching criteria and number of exposed and unexposed. Case-control study—for matched studies, give matching criteria and the number of controls per case.~~
- (RECORD) The methods of study population selection (such as codes or algorithms used to identify participants) should be listed in detail. If this is not possible, an explanation should be provided. Exposures, outcomes, and covariates
- (RECORD) Any validation studies of the codes or algorithms used to select the population should be referenced. If validation was conducted for this study and not published elsewhere, detailed methods and results should be provided. Exposures, outcomes, and covariates
- (RECORD) If the study involved linkage of databases, consider use of a flow diagram or other graphical display to demonstrate the data linkage process, including the number of individuals with linked data at each stage. eFigure 3
- (RECORD-PE) Describe the study entry criteria and the order in which these criteria were applied to identify the study population. Specify whether only users with a specific indication were included and whether patients were allowed to enter the study population once or if multiple entries were permitted. See explanatory document for guidance related to matched designs. Study population, Statistical analyses, Figure 1

7. Variables

- (STROBE) Clearly define all outcomes, exposures, predictors, potential confounders, and effect modifiers. Give diagnostic criteria, if applicable. Exposures, outcomes, and covariates
- (RECORD) A complete list of codes and algorithms used to classify exposures, outcomes, confounders, and effect modifiers should be provided. If these cannot be reported, an explanation should be provided. Exposures, outcomes, and covariates
- (RECORD) A complete list of codes and algorithms used to classify exposures, outcomes, confounders, and effect modifiers should be provided. If these cannot be reported, an explanation should be provided. Access
- (RECORD-PE) Describe how the drug exposure definition was developed. Exposures, outcomes, and covariates
- (RECORD-PE) Specify the data sources from which drug exposure information for individuals was obtained. Data sources
- (RECORD-PE) Describe the time window(s) during which an individual is considered exposed to the drug(s). The rationale for selecting a particular time window should be provided. The extent of potential left truncation or left censoring should be specified. Exposures, outcomes, and covariates
- (RECORD-PE) Justify how events are attributed to current, prior, ever, or cumulative drug exposure. Statistical analyses
- (RECORD-PE) When examining drug dose and risk attribution, describe how current, historical or time on therapy are considered. Exposures, outcomes, and covariates
- (RECORD-PE) Use of any comparator groups should be outlined and justified. Exposures, outcomes, and covariates
- (RECORD-PE) Outline the approach used to handle individuals with more than one relevant drug exposure during the study period. Exposures, outcomes, and covariates

8. Data sources/measurement

© 2023 Matthewman J et al. *JAMA Dermatology*.

- (STROBE) For each variable of interest, give sources of data and details of methods of assessment (measurement). Describe comparability of assessment methods if there is more than one group. Exposures, outcomes, and covariates
- (RECORD-PE) Describe the healthcare system and mechanisms for generating the drug exposure records. Specify the care setting in which the drug(s) of interest was prescribed. Data sources

9. Bias

- (STROBE) Describe any efforts to address potential sources of bias. Statistical analyses

10. Study size

- (STROBE) Explain how the study size was arrived at. Study population

11. Quantitative variables

- (STROBE) Explain how quantitative variables were handled in the analyses. If applicable, describe which groupings were chosen, and why. Exposures, outcomes, and covariates

12. Statistical methods/Data access and cleaning methods/Linkage

- (STROBE) Describe all statistical methods, including those used to control for confounding. Statistical analyses
- (STROBE) Describe any methods used to examine subgroups and interactions. Statistical analyses
- (STROBE) Explain how missing data were addressed. Exposures, outcomes, and covariates
- (STROBE) Cohort study—if applicable, explain how loss to follow-up was addressed. ~~Case-control study—if applicable, explain how matching of cases and controls was addressed. Cross sectional study—if applicable, describe analytical methods taking account of sampling strategy.~~ Section 2.2.5
- (STROBE) Describe any sensitivity analyses. Statistical analyses, Table 1
- (RECORD-PE) Describe the methods used to evaluate whether the assumptions have been met. Statistical analyses, Table 1
- (RECORD-PE) Describe and justify the use of multiple designs, design features, or analytical approaches. Statistical analyses, Table 1
- (RECORD) Authors should describe the extent to which the investigators had access to the database population used to create the study population. Appendix
- (RECORD) Authors should provide information on the data cleaning methods used in the study. Appendix
- (RECORD) State whether the study included person level, institutional level, or other data linkage across two or more databases. The methods of linkage and methods of linkage quality evaluation should be provided. Appendix

Results

13. Participants

- (STROBE) Report the numbers of individuals at each stage of the study (eg, numbers potentially eligible, examined for eligibility, confirmed eligible, included in the study, completing follow-up, and analysed). eFigure 3
- (STROBE) Give reasons for non-participation at each stage. eFigure 3
- (STROBE) Consider use of a flow diagram. eFigure 3
- (RECORD) Describe in detail the selection of the individuals included in the study (that is, study population selection) including filtering based on data quality, data availability, and linkage.

© 2023 Matthewman J et al. *JAMA Dermatology*.

The selection of included individuals can be described in the text or by means of the study flow diagram. eFigure 3

14. Descriptive data
- (STROBE) Give characteristics of study participants (eg, demographic, clinical, social) and information on exposures and potential confounders. Table 2
- (STROBE) Indicate the number of participants with missing data for each variable of interest. Table 2
- (STROBE) Cohort study—summarise follow-up time (eg, average and total amount). eTable 2

15. Outcome data
- (STROBE) Cohort study—report numbers of outcome events or summary measures over time. ~~Case-control study—report numbers in each exposure category, or summary measures of exposure. Cross sectional study—report numbers of outcome events or summary measures.~~ Table 3

16. Main results
- (STROBE) Give unadjusted estimates and, if applicable, confounder adjusted estimates and their precision (eg, 95% confidence intervals). Make clear which confounders were adjusted for and why they were included. Table 3
- (STROBE) Report category boundaries when continuous variables are categorised. Table 3
- (STROBE) If relevant, consider translating estimates of relative risk into absolute risk for a meaningful time period.

17. Other analyses
- (STROBE) Report other analyses done—eg, analyses of subgroups and interactions, and sensitivity analyses. Appendix

Discussion

18. Key results
- (STROBE) Summarise key results with reference to study objective. Discussion

19. Limitations
- (STROBE) Discuss limitations of the study, taking into account sources of potential bias or imprecision. Discuss both direction and magnitude of any potential bias. Discussion
- (RECORD) Discuss the implications of using data that were not created or collected to answer the specific research question(s). Include discussion of misclassification bias, unmeasured confounding, missing data, and changing eligibility over time, as they pertain to the study being reported. Discussion
- (RECORD-PE) Describe the degree to which the chosen database(s) adequately captures the drug exposure(s) of interest. Discussion

20. Interpretation
- (STROBE) Give a cautious overall interpretation of results considering objectives, limitations, multiplicity of analyses, results from similar studies, and other relevant evidence. Discussion
- (RECORD-PE) Discuss the potential for confounding by indication, contraindication or disease severity or selection bias (healthy adherer/sick stopper) as alternative explanations for the study findings when relevant. Discussion

21. Generalisability
- (STROBE) Discuss the generalisability (external validity) of the study results. Discussion

© 2023 Matthewman J et al. *JAMA Dermatology*.

Other information

22a. Funding/Accessibility of protocol, raw data, and programming code

- (STROBE) Give the source of funding and the role of the funders for the present study and, if applicable, for the original study on which the present article is based. Funding
- (RECORD) Authors should provide information on how to access any supplemental information such as the study protocol, raw data, or programming code. Access

© 2023 Matthewman J et al. *JAMA Dermatology*.

A.5 eReferences.

29 Clinical Practice Research Datalink. Small area level data based on patient postcode. 2022.

30 Wilkins R. Use of postal codes and addresses in the analysis of health data. *Health Rep* 1993;**5**:157–77.https://www.ncbi.nlm.nih.gov/pubmed/8292756

31 Glazier RH, Creatore MI, Agha MM, *et al.* Socioeconomic misclassification in ontario's health care registry. *Can J Public Health* 2003;**94**:140–3.https://www.ncbi.nlm.nih.gov/pubmed/12675172

32 Juurlink D, Preyra C, Croxford R, *et al.* Canadian institute for health information discharge abstract database: A validation study. 2006.

33 Williams J, Young W. A summary of studies on the quality of health care administrative databases in canada. *Patterns of health care in Ontario: the ICES practice atlas 2nd ed Ottawa: Canadian Medical Association* 1996;**339**:45.

34 Levy AR, O'Brien BJ, Sellors C, *et al.* Coding accuracy of administrative drug claims in the ontario drug benefit database. *Can J Clin Pharmacol* 2003;**10**:67–71.https://www.ncbi.nlm.nih.gov/pubmed/12879144

35 Ontario Ministry of Finance. Ontario Demographic Quarterly: Highlights of first quarter. 2021.http://www.ontario.ca/page/ontario-demographic-quarterly-highlights-first-quarter

36 Robles SC, Marrett LD, Clarke EA, *et al.* An application of capture-recapture methods to the estimation of completeness of cancer registration. *J Clin Epidemiol* 1988;**41**:495–501. doi:10.1016/0895-4356(88)90052-2

37 British National Formulary. Glucocorticoid therapy. https://bnf.nice.org.uk/treatment-summaries/glucocorticoid-therapy/

38 Andridge RR, Little RJA. A Review of Hot Deck Imputation for Survey Non-response. Int Stat Rev. 2010 Apr;78(1):40–64.

39 Langan SM, Schmidt SA, Wing K, *et al.* The reporting of studies conducted using observational routinely collected health data statement for pharmacoepidemiology (RECORD-PE). *BMJ* 2018;k3532. doi:10.1136/bmj.k3532

© 2023 Matthewman J et al. *JAMA Dermatology*.
