# Supplementary material for: Association of Different Prescribing Patterns for Oral Corticosteroids With Fracture Preventive Care Among Older Adults in the UK and Ontario
Source: JAMA Dermatol. 2023 Aug 9;159(9):961–9. doi: 10.1001/jamadermatol.2023.2495 (PMC10413212; doi:10.1001/jamadermatol.2023.2495)
Supplement: Supplement 2. — Data Sharing Statement [file jamadermatol-e232495-s002.pdf]

Data Sharing Statement

Matthewman. Association of Different Prescribing Patterns for Oral Corticosteroids With Fracture Preventive Care Among Older Adults in the UK and Ontario. *JAMA Dermatol*. Published August 09, 2023. doi:10.1001/jamadermatol.2023.2495

Data
Data available: No

Additional Information
Explanation for why data not available: Access to CPRD and ICES data is subject to respective approvals processes, therefore the authors are not able to grant access to the raw data used for this study. Access can be granted through the respective data provider and may incur a cost.
For the UK study, all analysis codes and code lists used for this study are available on zenodo.org under the doi 10.5281/zenodo.7950694. For the Ontario study, the full dataset creation plan and underlying analytic code are available from the authors upon request, understanding that the computer programs may rely on coding templates or macros that are unique to ICES and are therefore either inaccessible or may require modification.
